# Understanding genetic diversity and population genetic structure of three Cyprinidae fishes occupying the same habitat from Uttarakhand, India

**DOI:** 10.1080/23802359.2019.1662740

**Published:** 2019-09-09

**Authors:** Bheem Dutt Joshi, J. A. Johnson, Tarana Negi, Ashutosh Singh, S. P. Goyal, Ram Krishan Negi

**Affiliations:** a Department of Zoology and Environmental Sciences, Gurukula Kangri University, Haridwar, India;; b Wildlife Institute of India, Dehradun, India;; c Department of Zoology, Govt. College, Bahadurgarh, India;; d Department of Zoology, University of Delhi, Delhi, India

**Keywords:** *Puntius*, *Pethia*, phylogenetics, cryptic species, population structure

## Abstract

Different pattern of genetic diversity and population genetic structure among the species are reported due to their different ecological requirements, adaptability and the evolutionary histories. Understanding such patterns in a species and between the populations is important to develop the effective conservation plans. Very limited studies are available, how different factors influencing the gene flow of a species especially in fish communities. Therefore, the present study is aimed to document the genetic diversity and population genetic structure of the three species of Cyprinidae fishes (*Puntius sophore*, *Pethia ticto*, and *Pethia conchonius*) sharing the same kind of habitat using the mitochondrial cytochrome c oxidase subunit 1 (CO1). We used 80 samples of the three species from different river/streams. In which we observed total 4–9 haplotypes in all three species with the intra-species sequenced divergence ranges between 0.002 and 0.019. The nucleotide and haplotype diversity was ranged from 0.002040 to 0.01007 and from 0.251 to 0.822, respectively. Neutrality test values were found to be positive only in the *P. ticto* but statistically non-significant. The AMOVA variation among the populations was 8.89–84.30% whereas, within the populations, it was ranged from 15.70 to 91.11%. The median-joining haplotype network suggests the stable population size over the time and haplotypes were clustered with respect to their geographic locations except the *P. conchonius*. Similar pattern observed in the phylogenetic tree.

## Introduction

Revealed different pattern of genetic diversity of species those sharing sympatric habitat infer the different ecological requirements and the high adaptability to the diverse kind of the environment (Vass et al. 2010). However, understating different genetic parameter of the species and their evolutionary history is important for the species conservation. Where, the aquatic species are less evaluated specially for their genetics correlating with other ecological and environmental variables. The fishes of the genus *Puntius*/*Pethia* (Family Cyprinidae) are small size and have the beautiful colorations pattern make them popular as freshwater aquarium fishes and many species are traded internationally (Collins et al. 2012). The species of these two (*Pethia* and *Puntius*) are highly adaptive to different aquatic habitat such as streams, rivers, canals, lakes, reservoirs, and other wetlands. In India, the species of both the genus almost distributed throughout India except higher altitude of the Himalayan ranges (Talwar and Jhingran 1991). Apart from these areas, these species also distributed in the Bangladesh, Nepal, Pakistan, and Afghanistan and found in lakes and flowing waters in subtropical parts (Varadi and Horvath 1993). These species have been over harvested from their native habitat for food and aquarium trade.

The fishes that are prone to the over harvesting shown the declining population trend or restricted them in fragmented or highly specified habitat (Anon 1998; Dubut et al. 2012). Many threats identified for declining of fish population, such as deforestation, watershed erosion, siltation, agricultural runoffs, pesticides, fertilizers, sewage, and chemical pollutants (Ponniah and Gopalakrishnan 2000). All these contributing factors greatly affected fish biodiversity that leads to decline in fish catch, an apparent shift species composition, and an increasing occurrence of invasive fish species (Mishra et al. 2007; Vass et al. 2010). Such threats also may cause the genetic erosion through bottlenecks or founder effects during colonization (Hewitt 2000). This also leads in declining genetic diversity with increasing distance from source population (Grivet and Petit 2003; Eckert et al. 2008). Therefore, it is important to understand how demographic and selective processes interact at landscape level during colonization and affect the evolution of a species. Mitochondrial DNA marker is commonly used in both freshwater and marine fish species to document the genetic diversity and demographic history (Weersing and Toonen 2009). In this study, we evaluated the patter of the genetic diversity of three species of *Puntius sophore*, *Pethia ticto*, and *Pethia conchonius* that share the same habitat.

## Materials and methods

### Sample collection

Samples of the *Puntius/Pethia* species were collected from different tributaries of Ganga and Yamuna river in Uttarakhand state – Site-I Diowala, Dehradun Song River (30°18′N 78°13′E), Site-II, Vikasnagar, Asan River (30°43′N 77°72′E) and Site III, Kotdwar, Kho River (29°73′N 78°52′E). Samples were collected using different fishing gears *viz.*, hand net, cast net, and some were collected from fisherman and other indigenous trapping method during 2013–2014. Samples were identified up to species level in the field using the taxonomic key (Talwar and Jhingran 1991; Jayaram 2010). For the fish samples collection all the required permission were obtained from the Uttarkhand Forest Department with the letter No. vide No 2677/5–6 Dated 24.04.2014. All the specimens/tissue samples were deposited to the Wildlife Institute of India’s reference repository with sample ID for *P. sophore* (PSB1-B10, PSA1-A10, and PSD1-D10), *P. ticto* (PSB1-B10; PTD1-D10), and *P. conchonius* (PCA1-A10, PCB1-B10 and PCD1-D10).

### DNA extraction and PCR amplification

DNA was extracted from the 80 tissue samples (*P. sophore* (30), *P. ticto* (20), *P. conchonius* (30)) using DNeasy Blood & Tissue Kit (Qiagen, Hilden, Germany) and the partial fragment of cytochrome c oxidase subunit 1 (*CO1*) gene (Folmer et al. 1994) were used for PCR amplification of DNA extracted from the 80 tissue samples. Based on the quality of DNA extract, DNA was diluted with nuclease-free water at 1:100 and direct template for the DNA samples were used in those samples where the DNA was not visible on gel (Joshi et al. 2019). PCR amplification was carried out with DNA extract and the composition of PCR master mix for 10 µl reaction volume was 1 × PCR Buffer; 2 mM MgCl_2_; 200 µM dNTP; 0.4 µM of each primer; 0.5 U Taq polymerase (MBI, Fermentas), and 40 ng of genomic DNA. PCR thermal cycling parameters included initial denaturation at 94 °C for 5 min followed by 45 cycles of denaturation at 94 °C for 45 s, annealing at 45 °C for 1 min, and extension at 72 °C for 50 s with one cycle of a final extension for 20 min at 72 °C for the *CO1* gene. Amplification was verified on the 2% (w/v) agarose gel by loading a mixture of 4 μl PCR product and 1 μl loading dye. The bands of amplified product were observed under the UV light. Amplified PCR products were processed for the cycle sequencing followed by Exo-Sap (Exonuclease I-Srimp Alkaline Phosphatase) treatment to remove the residual primers and dNTPs prior to cycle sequencing PCR. PCR products were than cycle sequenced with their respective primers following the suggested composition of master mixture by manufacture (Applied Biosystems, Foster City, CA). These products then subjected to DNA sequencing on ABI 3130 genetic analyzer.

### Data analysis

#### Sequence editing, assembly, and alignment

Raw sequences of the *CO1* gene were examined and validated using Sequencher version 4.7 (Gene Codes Corporation, Ann Arbor, MI). The sequences generated in this study were validated using reference data through the BLAST tool of GenBank (http://www.ncbi.nlm.nih.gov) in which all the sequences were identified as the respective species. The sequences were aligned with the complete genome of *Pethia ticto* using Bioedit (Hall 1999). All the generated sequences were submitted to NCBI, Genbank of *P. conchonius* (Accession nos. KT957174–KT957188); *P. sophore* (KT957189–KT957196); *P. ticto* (KT957197–KT957203).

Polymorphic sites and haplotypes were identified using MEGA version 6 (Tamura et al. 2013), DnaSP version 5.10 (Librado and Rozas 2009), Bioedit (Hall 1999), and private or local haplotype were assessed through a visual inspection in Bioedit (Hall 1999). Sequence divergence was calculated using the Kimura 2 Parameters (K2P) distance matrix in the MEGA version 6 (Tamura et al. 2013). Nucleotide (*π*) and haplotype (*h*) diversity values were estimated with ARLEQUIN version 3.0 (Excoffier et al. 2007) and DnaSP version 5.10 (Librado and Rozas 2009) as these diversities influenced by past evolutionary and demographic history of the species. Neutrality test values were calculated using the DnaSP 5.10 (Librado and Rozas 2009) and ARLEQUIN 3.0 (Excoffier et al. 2007).

#### Phylogenetic and network analysis

Phylogenetic tree was constructed using different statistical methods e.g. neighbour-joining (NJ), maximum-likelihood (ML), and maximum-parsimony (MP) as executed in MEGA version 6 (Tamura et al. 2013) using 1000 bootstrap replicates. To understand the proximity of haplotypes based on genetic distance and variation between haplotypes in the sample, median-joining (MJ) networks were constructed using Network version 4.611 (Bandelt et al. 1999). Use of haplotype network to understand the pattern of haplotype distribution is more suitable than the use of phylogenetic trees, which assume the presence of ancestral haplotypes in the population (Clement et al. 2000). For calculating Network, the parameters were set as of Forster et al. (2001).

## Results

### Species identification, nucleotide variability, and genetic distance

We sequenced 650 bp of the *CO1* gene from 80 samples of three species – *P. sophore*, *P. ticto*, and *P. conchonius.* Out of 650 long nucleotide sequences, 550 bp sequences were found suitable for analysis after validation. The nucleotide frequency was observed between 9.4 and 46.1% for the ATGC (Table 1). In the *CO1* gene, total 11, 13, and 4 variable sites were observed, whereas 9, 8, and 4 haplotypes were observed in the *P. sophore*, *P. ticto*, and *P. conchonius*, respectively (Table 1). The sequence divergence between the haplotypes was observed ranges from 0.002 to 0.017 in *P. sophore*, 0.001–0.019 in *P. ticto*, and 0.002–0.009 in *P. conchonius*. In the *CO1* gene, four local (private) haplotype (Hap 4, 5, 6, and 8) were observed in samples of *P. sophore* collected from the Doiwala whereas in *P. ticto* only two sites were sampled, among which 8 haplotypes observed. In the *P. conchonius* only three haplotypes were observed.

**Table 1.** Nucleotide composition of *CO1* gene.

	T (%)	C (%)	A (%)	G (%)
*P. sophore*	31.2	25.2	27.0	16.5
*P. ticto*	31.6	24.6	27.0	16.7
*P. conchonius*	31.0	25.3	26.1	17.5

### Diversity indices, AMOVA, and demographic analysis

In all three species, the observed nucleotide diversity ranged from 0.00107 to 0.010953 and haplotype diversity ranged from 0.0251 to 0.9266 (Table 2). The AMOVA analysis revealed that among the population variation observed was 84.40% and within the population it was 15.60% in the *P. sophore*; (Table 3). *Pethia ticto* represented 8.89% variation among the population and 91.11% within the population, which were statistically significant. *Pethia conchonious*, represented 53.57% among the population variation where 46.43% within the population. In the case of neutrality test, the Tajima’s D values was −0.87397 in the *P. sophore*, 0.31147 in the *P. ticto* and −1.60513 in *P. conchonius.* The Fu’s *Fs* test values were −4.136 in *P. sophore*, 0.030 in the *P. ticto*, and −1.123 in the *P. conchonius* but none of these values were found statistically significant. The observed mismatch distribution graph was also supporting the results of neutrality test as multimodal pattern of all the three species.

**Table 2.** Diversity and neutrality indices in the *CO1* gene from the different river basin in Uttarakhand, India.

Species	*M*	*S*	*Nh*	*π*	*hd*	*D*	Fu’s *F*s	Fu and Li’s *D*
*P. sophore*								
Overall	30	36	8	0.010953	0.9266	−0.87397	−4.136	−1.94191
Site-A	10	5	5	0.002680	0.822	−1.03527	−1.587	−0.68600
Site-B	10	1	2	0.000410	0.200	−1.11173	−0.339	−1.24341
Site-D	10	5	2	0.002040	0.200	−1.74110*	2.197	−2.01007
*P. ticto*								
Overall	20	13	8	0.00847	0.837	0.31147	0.030	1.09797
Site-B	10	2	3	0.00118	0.511	−0.69098	−0.594	−0.28020
Site-D	11	11	5	0.01007	0.800	0.97309	1.426	0.79062
*P. conchonius*								
Over All	30	5	4	0.00107	0.251	−1.60513	−1.123	−0.60837
Site-A	10	2	2	0.00082	0.378	−1.40085	−1.164	−1.58662
Site-B	n/c							
Site-D	10	3	2	0.00219	0.356	0.02107	2.338	1.15417

M: number of sequences; S: number of segregating sites; *Nh*: number of haplotype; *hd*: haplotype diversity; π: nucleotide diversity; D: Tajima’s D test statistic. Site-A, Song river, Doiwala; Site-B, Asan river, Vikasnagar; Site-D, Kho river, Kotdwar.

**Table 3.** AMOVA analysis of two populations of *P. ticto*.

	Source of variation	Sum of squares	Variance components	Percentage of variation
*P. sophore*	Among the population	14.265	1.96889 Va	84.30
	Within the Population	5.500	0.36667 Vb	15.70
	Total	19.765	2.33556	100.00
*P. ticto*	Among the population	20.90	1.92	53.57
	Within the Population	30.00	1.66	46.43
	Total	50.90	3.59	100.0
*P. conchonius*	Among the population	1.20	0.029	8.89
	Within the Population	8.20	0.303	91.11
	Total	9.40	0.333	100.0

### Phylogenetic analysis

**Figure 1.**
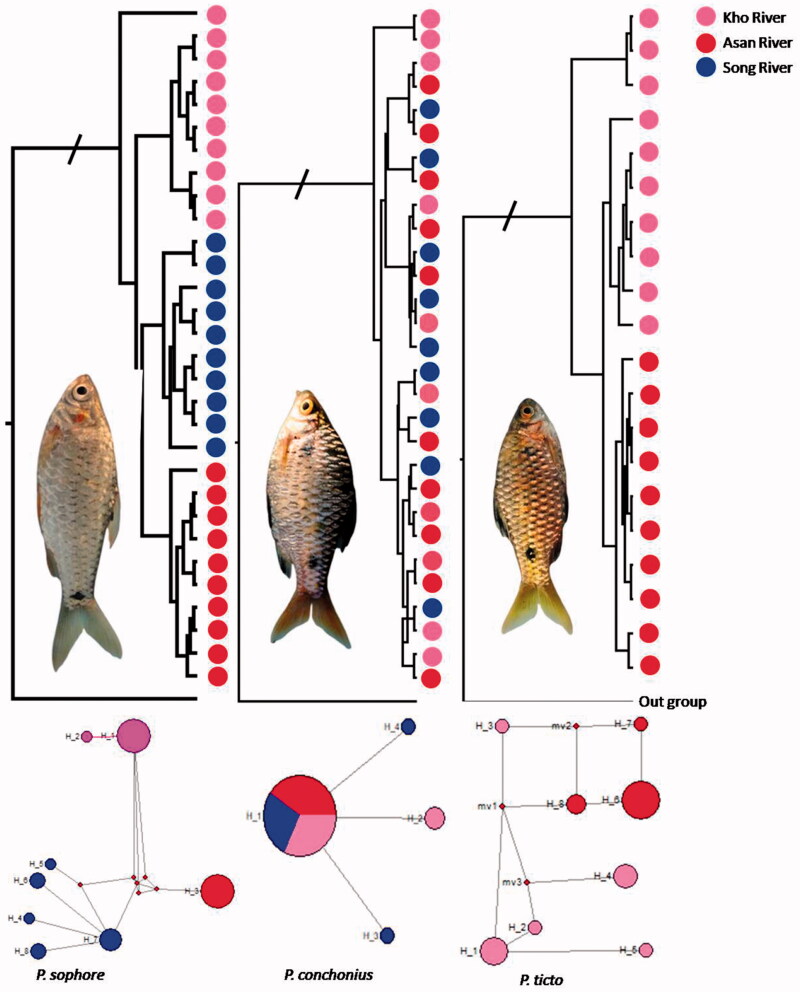
Phylogenetic tree and MJ network showing haplotype distribution from different river basin in Uttarakhand, India.

The results of phylogenetic tree analysis showed that *P. sophore* separated in three clades corresponding to their geographic locations/rivers with the similar cladistic pattern in the *P. ticto* which is only sampled from two sampling locations of Asan river and Kho river. On the other hand, all the samples of *P. conchonius* were clustered a single clade, irrespective to their geographic origin (Figure 1). In the M–J Network, only *P. conchonius* form the star-like topology, whereas the *P. sophore* and *P. ticto* shown the population genetic structuring (Figure 1). In the Network, the haplotype of *P. sophore* and *P. ticto* connected with median vectors, which suggest presence of the missing haplotypes.

## Discussion

The present investigation, a total of 9, 8, and 4 haplotypes were observed in *P. sophore, P. ticto*, and *P. conchonius*, respectively. The observed intra-species sequences divergence was ranges from 0.002 to 0.017 for *P. sophore* and 0.001–0.019 for *P. ticto*, and 0.002–0.009 for *P. conchonius.* The nucleotide and haplotype diversity was found high in the *P. ticto* compare to other two species (Table 2). In the respective sampling sites, the haplotype diversity was found high in site D (Kho river) for the *P. ticto* whereas in the *P. sophore* it was found high in site A (Song river). A total of five private haplotypes were observed in site A (Song river, Doiwala). One of the sequences from the Kotdwar found in the outer clade shows the high genetic distance (0.017) with the other samples of the *P. sophore*. Whereas for the *P. ticto* three samples are showing the genetic distance (0.019) and formed the separate clade (Figure 1) when we compare these sequences with the other NCBI sequences, these samples were clustered with *P. ticto* individuals, originated from Uttar Pradesh (Data not shown). These sequences are suggestive presence of cryptic species or species complex for the *P. ticto* in the same kind of the habitat. However, the diversity indices are observed all the studied fish species were similar as reported in the other studies of the different cyprinid fishes (nucleotide diversity 0.01346–0.0237; Sah et al. 2011) and was recorded as 0.0–0.1 (haplotype diversity 0.0–0.9; Esa et al. 2008; Sah et al. 2011) in India. In the other part of the world among the cyprinid fish, the nucleotide diversity was at moderate level which ranges from 0.0024 to 0.0045 (Qi et al. 2007). Whereas the nucleotide diversity was reported lower than the average value (0.0014–0.0020) in the few cyprinid fishes (Perdices et al. 2004, 2005). The results of haplotype diversity (0.937) and nucleotide diversity (0.0089) of *CO1* gene were in consistence with this study (Taillebois et al.^2013^). Both the neutrality test (Tajima’s *D* and Fu’ *Fs*) revealed that positive values were observed only for *P. ticto* from the Kho river, suggest the presence of hybrid or missing of the alleles in the gene pool. However, further confirmation study is needed using large samples size and loci. In the phylogenetic tree, all the samples from different locations formed separate clades; 3 for *P. sophore* and 2 for *P. ticto* (samples from only two location). Whereas for *P. conchonius* only single clade were formed irrespective to their geographic/river affinity (Figure 1). Such pattern observed in *P. conchonius* may be due to occurrence of incomplete lineage shorting in this gene (Ward 2009). On other hand, low haplotype frequency observed in *P. conchonius CO1* gene may also be due to slow evolutionary rate or this species may be highly adaptive to different ecological conditions (Lenski 2001). Another reason could be that occurrence of this species in almost all types of habitats such as wetlands, slow flowing channel unit, isolated pools, and secondary channel of water during the summer when river gets dried (per. Obs). This species also co-exists with other bottom dwelling species like *Schistura* live in seasonal streams and channel unit with low water. Such organisms have presumably run out of ways to become better adapted to their environment (Lenski 2001). MJ-haplotype network showed that the star-like topology was not formed in *P. ticto* and *P. sophore*, that indicates these populations have not gone under recent population expansion and showing long-term stability (Mila et al. 2000; Xue et al. 2014) as star-like topology is the result of population expansion. However, star-like topology observed for the *P. conchonius*, but other demographic test reject the population expansion.

## Conclusion

We have explored the genetic diversity and pattern of the population genetic structure in three species genus *Pethia/Puntius* using the *CO1* gene. These patterns indicate how the different species are adapted to same kind of the environment through exhibiting the different pattern of population genetic structuring, where two species show a sharp population genetic structuring signals, whereas other rejects. We also observed the private haplotype in the *P. sophore* in Song river as the water of this sparsely distributed and this river also having the high anthropogenic pressure. Results indicate *P. ticto*, shows the cryptic diversity and we recommend to use of large samples size and loci to delineate the species boundaries.

## Authors’ contributions

BDJ designed the study, carried out the samples collection, DNA extraction, Sequencing and data analysis, write the MS; JAJ provided the reagents and helped in the MS writing; TN has helped in analysis and MS writing; AS has compiled the MS performed data analysis; SPG has co-supervised the work edited MS and provided the Lab support. RKN conceived the study and supervised manuscript.

## Data Availability

Data are available on request as well as in the NCBI database. All required material and methods given in the Materials and Methods section.
